# Plasma IL-25 is elevated in a subgroup of patients with clinical reactivity to peanut

**DOI:** 10.1186/2045-7022-3-40

**Published:** 2013-12-02

**Authors:** Joost A Aalberse, Anders O van Thuijl, Yolanda Meijer, Wilco de Jager, Tjitske van der Palen-Merkus, Aline B Sprikkelman, Maarten O Hoekstra, Berent J Prakken, Femke van Wijk

**Affiliations:** 1 Department of General Paediatrics, University Medical Centre Utrecht, Wilhelmina Children’s Hospital, Utrecht, The Netherlands; 2 Laboratory for Translational Immunology, Department of Paediatric Immunology, University Medical Centre, PO BOX 85090, Utrecht 3508 AB, The Netherlands; 3 Department of Paediatric Respiratory Medicine and Allergy, Emma Children’s Hospital Academic Medical Center, Amsterdam, The Netherlands; 4 Department of Paediatrics, University Medical Centre Groningen, Groningen, The Netherlands; 5 Sanquin Research, Amsterdam, The Netherlands

**Keywords:** DBPCFC, IL-17 family, IL-25, Peanut allergy

## Abstract

**Background:**

One of the IL-17 family members, IL-25, has been implicated with the initiation and amplification of Th2 responses in animal models and has been associated with airway hyper-reactivity. The involvement of IL-25 and also IL-17 in food allergic disease remains to be investigated.

**Findings:**

In this study thirty children suspected of peanut allergic disease underwent a double-blind placebo controlled food challenge (DBPCFC) and IL-25 and IL-17 plasma levels were determined before and after challenge. IL-25 was highly elevated only in subgroup of children with a positive DBPCFC outcome. Plasma IL-25 was absent in children with a negative DBPCFC outcome and in healthy controls.

**Conclusions:**

This study shows that IL-25, an IL-17 family member, is highly elevated only in children with a clinical response to peanut. This suggests a role for IL-25 in the pathogenesis of peanut allergy and elevated plasma IL-25 may be a sign of a severe atopic phenotype.

## Findings

Members of the IL-17 cytokine family are emerging as key factors in immune responses [1]. The prototypic family member, IL-17A, triggers pro-inflammatory immune responses and contributes to neutrophilia during chronic airway inflammation. IL-17E, also known as IL-25, is the most divergent cytokine in the IL-17 family and, unlike the other members, has been identified as a central player in the initiation and amplification of Th2 responses [1]. In experimental mouse models IL-25 mediates early differentiation towards a Th2 phenotype and development of airway hyper-reactivity and allergic disease [2,3]. Moreover, allergen provocation in asthmatic patients increases expression of IL-25 and its receptor [4], suggesting that IL-25 is implicated in both sensitization and memory responses in airway hypersensitivity. Increased duodenal levels of Il-25 was found in peanut allergy in a mouse study [5]. In human subjects however, the involvement of IL-25 and IL-17 in food (peanut) allergy remains unknown. To investigate if there is a difference in IL-25- and IL-17 expression in peanut allergic versus peanut tolerant (i.e. peanut sensitized but, not clinical reactive) we determined IL-25 and IL-17 plasma levels, as well as the Th2 cytokines IL-4, IL-5 and IL-13, in a well-defined cohort of peanut sensitized children undergoing a double-blind placebo controlled food challenge (DBPCFC).

Thirty children suspected of peanut allergic disease (based on either elevated specific IgE to peanut (ImmunoCap >0.35 kU/L) or positive skin test to peanut) were referred to The Wilhelmina Children’s Hospital, University Medical Center, Utrecht, The Netherlands for a DBPCFC to obtain certainty about the diagnosis of peanut allergy (for patient characteristics see Table 1). The study was approved by the local medical ethics review boards (METC, UMC Utrecht; project no. 05/084 and METC AMC, Amsterdam; project no. 05/254) and informed consent was obtained for all subjects. The oral challenge was performed as previously described[6]. Peripheral blood samples were collected before the start of the DBPCFC, as well as when the challenge was finished. Plasma cytokine levels were determined by Xmap technology (Luminex Austin) [7].

**Table 1:** Characteristics peanut sensitized patients that underwent a DBPCFC

**No**	**Sex**	**Age**	**Peanut-specific-IgE at diagnosis**	**SPT at diagnosis**	**Asthma**^**#**^	**Atopic Dermatitis**^**$**^	**Clinical response to DBPCFC**	**GI**	**Resp**	**Syst**
		**(years)**								
**1**	**M**	**10**	**4.0**	**<1**	**-**	**+**	**-**			
**2**	**M**	**15**	**0.43**	**<1**	**+**	**+**	**-**			
**3**	**M**	**16**	**<0.35**	**3**	**-**	**-**	**-**			
**4**	**M**	**14**	**2.0**	**2**	**-**	**-**	**-**			
**5**	**M**	**4**	**5.9**	**NP**	**-**	**-**	**-**			
**6**	**F**	**3**	**0.6**	**3**	**-**	**+**	**-**			
**7**	**M**	**13**	**1.1**	**2**	**-**	**+**	**-**			
**8**	**M**	**4**	**7.3**	**<1**	**+**	**+**	**-**			
**9**	**M**	**15**	**0.6**	**2**	**-**	**+**	**-**			
**10**	**M**	**4**	**97.0**	**4**	**+**	**+**	**-**			
**11**	**M**	**8**	**0.8**	**3**	**-**	**-**	**-**			
**12**	**F**	**4**	**1.1**	**2**	**+**	**+**	**-**			
**13**	**M**	**5**	**>100**	**4.5**	**-**	**+**	**+**	**+**	**+**	**+**
**14**	**M**	**9**	**2.2**	**1**	**+**	**-**	**+**	*	*	*
15*	**F**	**6**	**>100**	**3**	**-**	**-**	**+**	**+**	**+**	**+**
16*	**M**	**17**	**56.0**	**3.5**	**-**	**+**	**+**	**-**	**+**	**-**
**17**	**M**	**6**	**>100**	**4**	**+**	**-**	**+**	**+**	**-**	**-**
**18**	**F**	**7**	**1.9**	**4**	**+**	**+**	**+**	**+**	**+**	**-**
**19**	**M**	**4**	**3.5**	**NP**	**-**	**+**	**+**	**+**	**+**	**+**
**20**	**M**	**9**	**>100**	**3**	**+**	**-**	**+**	**+**	**+**	**+**
**21**	**M**	**7**	**55.0**	**3**	**+**	**+**	**+**	**+**	**-**	**-**
**22**	**F**	**7**	**2.1**	**4**	**+**	**+**	**+**	**+**	**+**	**+**
23*	**M**	**11**	**4.0**	**2**	**-**	**+**	**+**	**+**	**-**	**+**
**24**	**M**	**4**	**6.0**	**2**	**-**	**+**	**+**	**+**	**+**	**-**
25*	**F**	**6**	**4.9**	**2**	**-**	**+**	**+**	**+**	**+**	**+**
**26**	**M**	**7**	**15.5**	**3**	**-**	**-**	**+**	**+**	**-**	**+**
27*	**M**	**5**	**21.0**	**2**	**+**	**-**	**+**	**+**	**+**	**-**
**28**	**F**	**6**	**5.3**	**3**	**+**	**+**	**+**	**+**	**-**	**-**
29*	**M**	**6**	**2.2**	**NP**	**+**	**+**	**+**	**+**	**+**	**+**
**30**	**F**	**5**	**NP**	**2**	**-**	**+**	**+**	**-**	**+**	**-**

Patient no. 1–12: non-responders to peanut challenge. Patient no. 13–30: positive responders to peanut challenge. ^#^ Asthma diagnosed according to ATS criteria.

**Figure 1:**
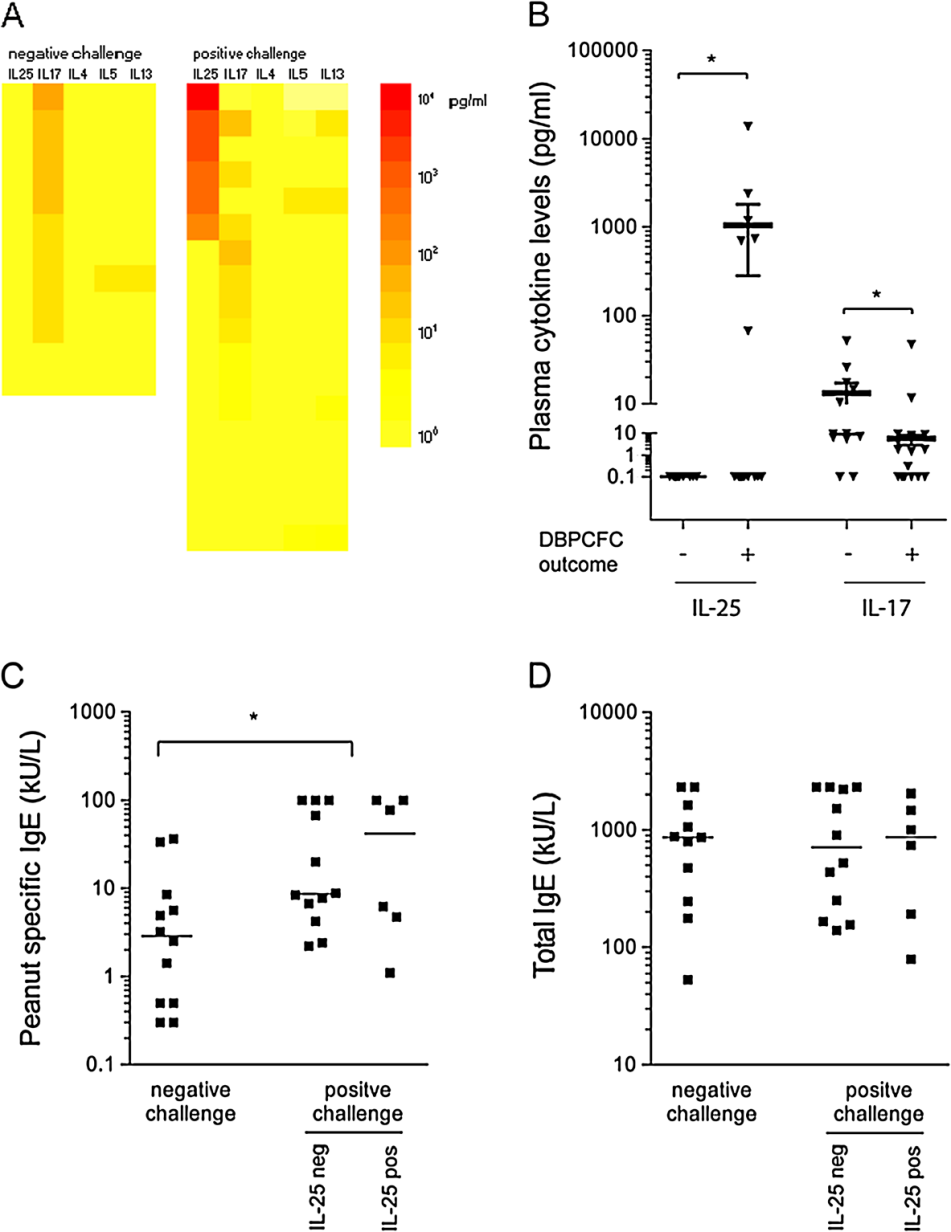
Cytokine profile and antibody levels of peanut sensitised patients*.* A colour profile **(A)** and scatter plot **(B)** of IL-25 and IL-17 plasma levels (pg/ml) in children with a negative (n = 12) and children with a positive peanut challenge (n = 18). * p < 0.05 with a Mann–Whitney U test. Peanut-specific IgE **(C)** and total IgE **(D)** serum levels in the negative and positive challenge groups,* p < 0.05 with a Mann–Whitney U test. The positive challenge group is subdivided in IL-25 positive and IL-25 negative children.

^$^Atopic dermatitis diagnosed according to criteria from Hanifin and Rajka. SPT, Skin Prick Test (the number represents the wheal size ratio peanut compared to histamine). DBPCFC, Double-Blind Placebo-Controlled Food Challenge. NP, Not performed. *Children with elevated plasma IL-25 as determined in Figure 1.

GI: Gastrointestinal symptoms including stomach ache, nausea, vomiting and diarrhea; Resp: respiratory symptoms including rhinorrhea, stuffy nose; Syst: systemic symptoms including shortage of breath, systemic urticaria and angioedema.

DBPCFC resulted in a positive (allergic) and negative (tolerant) challenge group and peanut allergy was confirmed in 18 out of 30 patients. In the plasma samples of the negative and positive challenge groups we found a striking difference for the type 2-related cytokine IL-25. Plasma IL-25 was not detected in any of the children with a negative challenge response, whereas plasma IL-25 was elevated in six children of the positive challenge group (Figure 1A). In five out of six of these children the IL-25 concentration was even extremely elevated, with levels ranging up to 13000 pg/ml (Figure 1A, B). IL-25 levels were similar in plasma samples taken after challenge (data not shown). IL-17 was found in both the positive and negative challenge groups (in 67% versus 83% of children respectively), but in contrast to IL-25, IL-17 levels were significantly lower in the positive challenge than in the negative challenge group (p < 0.01) (Figure 1A, B). We show that in this cohort of peanut sensitized children, the presence of high levels of IL-25 in plasma is only present in children with a positive challenge and clinical reactivity is inversely correlated with plasma IL-17. These findings seem to be rather specific for the cohort of (peanut) allergic children since both IL-25 and IL-17 plasma levels were below the detection limit in age-matched healthy controls (n = 20) (data not shown).

We searched to explain why plasma IL-25 was elevated in this subgroup of peanut allergic patients. No difference was found in relation to the other Th2 cytokines. Levels of IL-4 were below the detection limit and IL-5 and IL-13 levels were very low when positive. Within the clinical responder group also no difference in peanut-specific IgE levels was found between the IL-25 negative and IL-25 positive subgroups (Figure 1C).

Plasma IL-25 did not associate with total IgE levels (Figure 1D), previous exposure to peanut, severity of food allergic symptoms, or the presence of asthma and atopic dermatitis, nor did it correlate with organ specific symptoms during challenge (Table 1). As IL-25 is often mentioned to be related to asthma like symptoms we reviewed IgE data to inhalant allergens. Plasma levels of IgE to either birch, house dust mite (hdm) and cat from 17 patients were available. In general most children with a peanut sensitization were also sensitized to inhalation allergens, irrespective of the positive or negative challenge or IL-25 levels .

To extend our findings in peanut allergic children we next measured IL-17 and IL-25 in a group of infants diagnosed with cow’s milk allergy (CMA) (confirmed by DBPCFC, n = 12). In these infants IL-25 was found in 42% of the allergic patients but at very low levels (1.5-14 pg/ml) and IL-17 levels were below the detection level (data not shown). These data indicate that elevated IL-25 in serum is not a general phenomenon in clinical food allergy.

Peanut allergy is considered as an indication of a broad and possibly severe atopic phenotype and, unlike other food allergies (such as CMA), is infrequently outgrown [8]. The original diagnosis of peanut allergy in our tested cohort was not based on an oral challenge and this poses limitations on conclusions about the resolution of peanut allergy. However, the data do demonstrate that plasma IL-25 was only present in children with ongoing peanut allergy and, importantly, despite a peanut-free diet for at least 6 months. Together these data may indicate that elevated plasma IL-25 is a sign of chronic immune activation that is not induced by the provoking allergen itself but represents a risk factor for the development or persistence of clinical reactivity to peanut. Recently it was suggested that IL-25 secretion is induced during disruption of epithelial barriers. Our data are insufficient to make a conclusion on the nature of a IL-25 subgroup, but it can be speculated that these patients have a more severe type of food allergy. The finding that only 6 out of the 18 peanut allergic patients displayed highly elevated IL-25 levels stresses the possibility of a clinical subgroup within the group of peanut allergic children and warrants larger cohort studies.

IL-25 is expressed by a variety of innate immune cells and non-hematopoietic cells including basophils, eosinophils, epithelial and endothelial cells [9] and in the gut IL-25 is predominantly found in epithelial cells [10]. Innate lymphoid cells have been described in the human that respond to IL-25 and provide an innate source of Th2 cytokines [11,12]. Together, increased production of IL-25 triggered by environmental antigens or microbes in the gut might therefore contribute to the atopic phenotype by promoting Th2 differentiation and the maintenance of allergen specific Th2 memory cells.

In conclusion, this study is the first to show that IL-25 is highly elevated in a subgroup of peanut-allergic children and suggests a role for IL-25 in the development and/or persistence of peanut allergy, in this subgroup. These findings warrant further studies in a larger cohort of patients as well as in other food allergies.

## Abbreviations

DBPCFC: Double blind placebo controlled food challenge; CMA: Cow's milk allergy.

## Competing interests

The authors declare that they have no competing interests.

## Authors’ contributions

JA, MH, FvW, and BP designed research. YM, MH, and AS performed patient selection and DBPCFC. JA, WJ, TP, and AT performed experiments. JA, WJ, AT, and FvW analyzed and interpreted the data. JA and FvW wrote the paper. All authors read and approved the final manuscript.
